# Supplementary figures and images for: Password authenticated key exchange-based on Kyber for mobile devices
Source: PeerJ Comput Sci. 2024 Mar 29;10:e1960. doi: 10.7717/peerj-cs.1960 (PMC11042030; doi:10.7717/peerj-cs.1960)

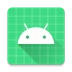

Supplement: Supplemental Information 1 [file peerj-cs-10-1960-s001.zip › kyberPAKE-raw-data/Kyber-PAKE-Java/Kyber-PAKE-Mobile-main/app/src/main/res/mipmap-hdpi/ic_launcher.webp]

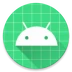

Supplement: Supplemental Information 1 [file peerj-cs-10-1960-s001.zip › kyberPAKE-raw-data/Kyber-PAKE-Java/Kyber-PAKE-Mobile-main/app/src/main/res/mipmap-hdpi/ic_launcher_round.webp]

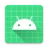

Supplement: Supplemental Information 1 [file peerj-cs-10-1960-s001.zip › kyberPAKE-raw-data/Kyber-PAKE-Java/Kyber-PAKE-Mobile-main/app/src/main/res/mipmap-mdpi/ic_launcher.webp]

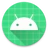

Supplement: Supplemental Information 1 [file peerj-cs-10-1960-s001.zip › kyberPAKE-raw-data/Kyber-PAKE-Java/Kyber-PAKE-Mobile-main/app/src/main/res/mipmap-mdpi/ic_launcher_round.webp]

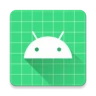

Supplement: Supplemental Information 1 [file peerj-cs-10-1960-s001.zip › kyberPAKE-raw-data/Kyber-PAKE-Java/Kyber-PAKE-Mobile-main/app/src/main/res/mipmap-xhdpi/ic_launcher.webp]

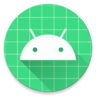

Supplement: Supplemental Information 1 [file peerj-cs-10-1960-s001.zip › kyberPAKE-raw-data/Kyber-PAKE-Java/Kyber-PAKE-Mobile-main/app/src/main/res/mipmap-xhdpi/ic_launcher_round.webp]

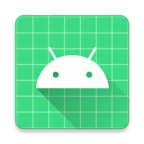

Supplement: Supplemental Information 1 [file peerj-cs-10-1960-s001.zip › kyberPAKE-raw-data/Kyber-PAKE-Java/Kyber-PAKE-Mobile-main/app/src/main/res/mipmap-xxhdpi/ic_launcher.webp]

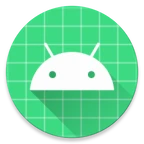

Supplement: Supplemental Information 1 [file peerj-cs-10-1960-s001.zip › kyberPAKE-raw-data/Kyber-PAKE-Java/Kyber-PAKE-Mobile-main/app/src/main/res/mipmap-xxhdpi/ic_launcher_round.webp]

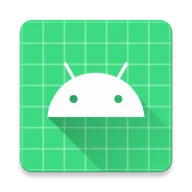

Supplement: Supplemental Information 1 [file peerj-cs-10-1960-s001.zip › kyberPAKE-raw-data/Kyber-PAKE-Java/Kyber-PAKE-Mobile-main/app/src/main/res/mipmap-xxxhdpi/ic_launcher.webp]

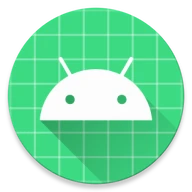

Supplement: Supplemental Information 1 [file peerj-cs-10-1960-s001.zip › kyberPAKE-raw-data/Kyber-PAKE-Java/Kyber-PAKE-Mobile-main/app/src/main/res/mipmap-xxxhdpi/ic_launcher_round.webp]
